# Quality-of-Life Evaluation among the Oldest-Old in China under the “Active Aging Framework”

**DOI:** 10.3390/ijerph19084572

**Published:** 2022-04-11

**Authors:** Xin Xu, Yuan Zhao, Jianfang Zhou, Siyou Xia

**Affiliations:** 1 Population Research Institute, Nanjing University of Posts and Telecommunications, Nanjing 210042, China; xuxin199010@126.com (X.X.); zhoujianfang@njupt.edu.cn (J.Z.); 2 Ginling College, Nanjing Normal University, Nanjing 210097, China; 3 Key Laboratory of Regional Sustainable Development Modeling, Institute of Geographic Sciences and Natural Resources Research, Chinese Academy of Sciences, Beijing 100101, China

**Keywords:** active aging framework, oldest-old, quality-of-life evaluation, China

## Abstract

China is facing an increasingly contradictory challenge between growing demand for health services for the oldest-old and the unbalanced and inadequate development in the context of rapid population aging. This study sought to evaluate the quality of life of the oldest-old in China under the active aging framework. Health, participation, and security data were sourced from China Statistics/Labor Statistics/Civil Affairs Yearbook 2000–2016 and National 1% Sample Survey Data 2005–2015. Then, we used the current life table, entropy method, coefficient variation, and panel data regression to evaluate the quality of life among the oldest-old and reveal its regional differences and mechanisms. The results show: (1) From 2005 to 2015, the overall quality of life in China steadily improved, and the quality of health, participation, and security of the oldest-old increased by 6.06%, 5.64%, and 47.48%, respectively. (2) Distinct regional disparities exist in the distribution of quality of life for the oldest-old in China; the “east–northeast–middle–west” stepped-declining pattern existed stably. (3) Population and family structure, economic development, and social security were the main reasons for the regional differences in quality of life for the oldest-old. Narrowing the socioeconomic gap between regions, promoting the function of family pension, and improving social old-age service supply will help improve the quality of life of the oldest-old.

## 1. Introduction

With the growing aging population and the socioeconomic development in China, properly solving the contradiction between the increasing demand for elder care services and the unbalanced and insufficient development of these services and improving quality of life (QoL) of the elderly have become important issues in scientific research in the new era [1]. Since the 18th National Congress of the Communist Party of China, the development of China’s aging industry has moved from focusing on the old-age care model to focusing on improving the QoL. In May 2016, General Secretary Xi Jinping explained the importance of improving the QoL of older adults to aid them in effectively coping with aging, and the construction of a system became part of the collective speech of the Political Bureau of the Central Committee [2]. In fact, in addition to the rapid expansion of older adults, the substantial increase in the proportion of the oldest-old has also become a source of pressure in China. According to authoritative forecasts, the population aged 80 and above in China will exceed 100 million in 2050, accounting for 30% of older adults. A large base, rapid growth, and poor overall health are the main characteristics of the population aging at this stage [3]. Under the premise of emphasizing “health” and “longevity”, how to measure and improve the QoL of the oldest-old in different regions has important theoretical and practical significance for improving the social policy system and creating expectations for a better life for the oldest-old [4].

Quality of life means comfortable life, convenient service facilities, spiritual enjoyment, and fun. Its concept originated in the United States in the 1930s [5]. After mature development in the 1950s and 1960s, QoL gradually became an objective or subjective indicator in the field of social research to measure welfare performance and psychological perception [6]. In the late 1970s, research on QoL expanded to the medical field. Compared with single indicators such as cure rate and survival rate of diseases, QoL better reflected the true state of physical, psychological, and social wellbeing. The concept also addressed changes in health concepts and medical models triggered by the disease spectrum [7]. The current foreign studies on the QoL of older adults mainly include the following aspects: ① Concept differentiation and theoretical explorations consensus, and integration and unification of opposing subjective and objective findings [8]. ② Scale evaluation and measurement with a focus on the individual (such as the 36-item short list of medical effects research, SF-36; European QoL Scale, EQ-5D; World Health Organization QoL Scale, WHOQOL-100) and group-scale development and cultural adjustment (Human Development Index, HDI) [9,10]. ③ Element association and mechanism analyses that focus on social psychology, cognitive function, leisure activities, living arrangements, and long-term nursing [11,12].

Domestic research on the QoL of the oldest-old began at the end of the 20th century. Early scholars qualitatively revealed the living conditions of the oldest-old in different regions through social surveys based on questionnaires. Since publication of “The Chinese Longitudinal Healthy Longevity Survey” (CLHLS) in 1998, research on the QoL of the oldest-old has shifted to a quantitative change. Physical health, mental health, social integrity, personality, and cognitive function related to QoL have begun to emerge, enriching the existing theory and method system [13,14]. In addition, centenarians, a special group of older adults, have attracted continuous attention from the academic community and will become a new entry point for analyzing and solving the health problems and longevity of older adults [15].

In general, the multidimensionality of QoL determines the dynamics of its measurement standards. At the same time, QoL has strong regional characteristics, not only in the differences in geographic location but also by regional value systems and cultural ideologies. Therefore, revealing the regional differences in QoL from the macro perspective, summarizing the development models of QoL in different regions, and looking for shortcomings that restrict the objective welfare conditions in the current construction of a harmonious society have become an important basis for guiding the improvement of QoL. However, existing studies focus on assessing the QoL from a single element, such as health quality, social participation, or pension services, artificially separating the internal logic of each element. Empirical experience and theoretical accumulation need to be further developed and improved. Studies based on CLHLS and other microsurvey data have become mature, and the QoL of groups measured from the geographical-space perspective is slightly weak. There are differences in economic base, resource endowment, social security, and human capital among the 31 provinces, autonomous regions, and municipalities directly under the central government in mainland China, and their QoL shows significant regional differences. As the oldest-old are vulnerable and high-risk groups for health issues, there is a need for more research on the impact of their living environment from a geographic perspective.

“Active Aging” refers to a policy aimed at improving the QoL of older adults and creating the best opportunities for health, participation, and security. It not only emphasizes the opportunity to participate in society at the individual level but also the process in which the rights and interests of older adults are guaranteed [16]. On 25 January 2017, the State Council issued the National Population Development Plan (2016–2030) to implement Active Aging, prioritizing the old-age service system, social security, and long-term care insurance. At the policy level, the combination of individual and national active aging is a theoretical innovation and manifestation of “Active Aging” in China, which indicates that the government is working hard to find a way to develop old-age care that is suitable for national conditions. This paper attempts to start from the perspective of the Active Aging policy framework, relying on relevant statistical data from 2005, 2010, and 2015, to comprehensively measure the QoL of the oldest-old in China to improve the understanding of regional differences in QoL and provide assistance in formulating policies to improve and coordinate the regional QoL for the oldest-old.

## 2. Theoretical Framework and Methods

### 2.1. Theoretical Framework

**Figure 1:**
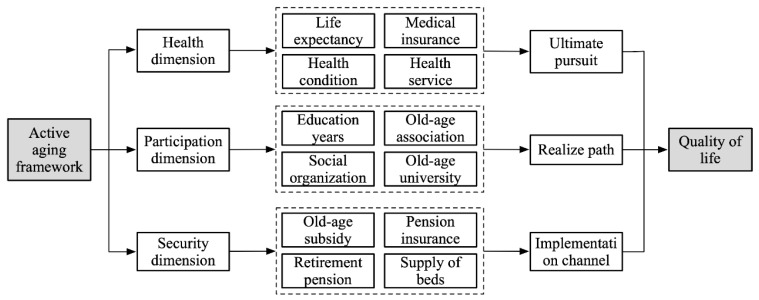
Theoretical framework of quality of life.

Our understanding of and response to aging has undergone a transition from “successful aging” to “healthy aging” and now to “active aging” [16]. In 2002, the second United Nations World Congress on Aging formally put forward Active Aging as a policy framework for the response to aging in the 21st century and wrote it into the “Political Declaration” [17]. Different from successful aging and healthy aging, active aging has shifted from a demand-based to a power-based focus that emphasizes the optimization of the health, participation, and security of older adults in an effort to prolong healthy life and improve QoL [16]. The connotation of active aging includes three dimensions: health, participation, and security (Figure 1).

(1) Health dimension. Health means reducing the chronic diseases caused by aging to extend the time that older adults can enjoy social participation, which is a fundamental pursuit of active aging. As a sensitive indicator of the QoL of older adults, health status could not only reflect the healthy living conditions of older adults directly but could also indirectly reflect their objective living conditions and the similarities and differences in life satisfaction caused by health differences that affect their QoL and well-being later in life [18,19]. The high prevalence of disabilities and loss of life partnership make the health of the oldest-old an urgent concern. Therefore, prioritizing equal access to medical resources, timely care, nursing, and rehabilitation when self-care ability is low are universal needs among the oldest-old [18].
(2) Participation dimension. Participation means that older adults participate in economic, social, cultural, and spiritual activities based on their own needs, preferences and abilities; this is the path to active aging. Social participation is an effective measure of how well older adults maintain social relationships, reshape social roles, and realize social identities [20]. “Activity Theory” and “Social Role Theory” contend that older adults who participate in social activities have higher life satisfaction and stronger social adaptability, which is beneficial to older adults in maintaining longevity and mental health in later life [21]. Studies have shown that social participation not only reduces the risk of death significantly, avoids disability before death, effectively relieves physical-function decline, reduces disease incidence, and improves health, but also can alleviate physical and mental degradation and reduce the incidence of Alzheimer’s disease, loneliness, and sense of loss [22]. At the same time, social participation is closely associated with education level. Older adults with a high level of education are more economically independent, have a lower burden of living, can calmly face and objectively analyze social and family problems, and have stronger adaptability and psychological adjustment capabilities. Older adults with higher education levels can also learn and communicate through books, newspapers, and other media and participate in activities at universities, activity centers, and associations for older adults to increase their life experience and improve their psychological well-being [23].
(3) Security dimension. Security means providing timely care for older adults in need of assistance from families, communities, and society in dealing with various risks and protecting their individual rights and interests. It is the implementation channel for active aging [16]. At present, the social security system, the old age allowance, the retirement payment system, the endowment insurance system, and the minimum living security system constitute the social system to ensure the economic support of older adults in China [24]. Due to their special physical, psychological, and family social support conditions, the urgent need of the oldest-old for social security manifested in two aspects: one is the need for a pension income. The oldest-old individuals were more born before liberation, and their wage and welfare benefits were relatively low. Although the social security system has incorporated them into the system after the reform and opening up, there are still many insecure older adults in urban and rural areas [25]. The second is the demand for old-age facilities. At present, the oldest-old mainly rely on home care. With the increasing weakening of family functioning, this old-age care method rooted in traditional Chinese culture is being challenged, and diversified old-age care methods are imperative [24].

### 2.2. Evaluation Index System

Based on the theoretical framework of active aging constructed above, we constructed the QoL evaluation index system of the oldest-old in three dimensions: health, participation, and security (Table 1).

Among them, the health dimension is evaluated by six indicators: average life expectancy (ALE), disability rate of older adults (DR), spouse rate of older adults (SR), participation rate of basic medical insurance for urban employees (UBMI), and number of medical and health technical personnel per 100 oldest-old (PMHT). The participation dimension consists of five indicators: the average years of education of older adults (AYE), the number of old-age social activity centers per 1000 older adults (SAC), the number of old-age organizations per 1000 older adults (OAO), the number of geriatric associations per 10,000 older adults (OAA), and the old-age school enrollment rate of older adults (OAS). The security dimension includes six indicators: the old-age subsidy coverage rate (OSC), the coverage rate of older adults’ retirement pension (PCR), the participation rate of basic pension insurance for urban employees (UBPI), the number of old-age service beds per 1000 oldest-old (BOC), the urban employees’ basic pension insurance benefits (BPB), and the socialized pension payment rate (SPP).

### 2.3. Influencing Factors Selection and Assumptions

The spatial differentiation of the QoL of the oldest-old are affected by many factors such as population, family, society, and economy. Drawing on existing studies [11], this research determined that the factors affecting the regional differences in the QoL of the oldest-old in China are “population and family structure, economic development, and social security”. The selection of relevant indicators based on the following assumptions:

(1) Population and family structure. The process of increasing the proportion of the oldest-old not only reflects the degree of healthy aging in the group but also the overall health status of older adults in the regions and the new longevity phenomenon [26]. This study found that before major breakthroughs in medicine, aging brought about a decline in the health of the oldest-old. Considering that China’s rapid socioeconomic development in recent years has provided the material basis for the extension of healthy life for older adults [27], we assumed that the higher the proportion of the oldest-old is, the better the QoL in a region. The old-age dependency ratio has an impact on residents’ consumption [28]. This study showed that changes in the demographic structure of a region, such as an increase in the proportion of the older adults, will increase the region’s consumption propensity for medical care, health care, and services, then improve the quality of life in the region [29]. Therefore, we assumed that the higher the old-age dependency ratio in the region is, the better the QoL. Family size is negatively associated with education and the economy significantly, and the miniaturization of family size has a positive impact on marriage and family life. At the same time, it can improve the quality of family life by reducing economic expenses. Therefore, we assumed that the smaller the average household size in the region, the better the QoL [30]. Based on the above assumptions, this paper selects the proportion of the oldest-old in older adults (POO), the old-age dependency ratio (ODR), and the average household size (AHS) to characterize the family structure of the population to analyze the impact on the QoL of the oldest-old.
(2) Economic development. Per capita GDP not only reflects the level and speed of regional development, but also includes the connotation of social equality, which constitutes the material basis for the income level of residents. The proportion of the output value of the secondary and tertiary industries in GDP has the dual nature of sociology and economics. It determines the employment structure of the labor force and measures the regional economic strength, the degree of modernization, and quality of people’s livelihoods. The level of urbanization is closely associated with the quality of material life, the availability of material resources, and spiritual resources. Therefore, this study uses per capita gross domestic product (GDP), the proportion of the output value of secondary and tertiary industries in GDP (PST), and urbanization level (UL) to represent the level of economic development to analyze the impact on the QoL of the oldest-old in China. At the same time, it assumed that the per capita GDP, the proportion of output value of secondary and tertiary industries in GDP, and the urbanization level and the QoL are significantly positively associated.
(3) Social security. As an important social function of the government, social security is closely associated with residents’ health. Social security enables older adults to share the fruits of economic development with dignity and realize the implementation of the quasi-pension policy [31]. Education level can reflect the state’s protection of citizens’ rights to education and has an important impact on the QoL of older adults. Considering that China’s existing social security system includes insurance, assistance, special care, and social welfare, the unemployment rate (UR) and social assistance expenditure per capita (SAE) are selected as the representations. With reference to existing research [32], the per capita years of education (AYE) is included in the social security category, and we assumed that per capita social assistance expenditure, unemployment rate, and the QoL of the oldest-old have a significant positive correlation, and per capita education level and QoL have a significant positive correlation.

### 2.4. Methods and Data Sources

#### 2.4.1. Current Life Table

A life table is a statistical table compiled according to the mortality rate of a specific age group, and used to compare and evaluate social health status, and to study the health level, characteristics, and changing rules of the population. The life table is divided into a current life table and a fixed group life table. Considering that the existing census data do not include the average life expectancy of provincial administrative units, this paper uses the current life table to calculate this indicator. The life table calculates a series of indicators such as the “death probability”, “the number of deaths”, “the number of survivors”, and “life expectancy” at ages along a trajectory for a generation born at the same time (generally 100,000) [33].

#### 2.4.2. Multi-Index Comprehensive Evaluation

First, to eliminate the dimensional influence between each index, each index is standardized. The positive index adopts the maximum effect standardization, and the reverse index adopts the minimum effect standardization principle. The calculation formulas are as follows:

(1) $$\mathrm{Positive}\mathrm{indicator}:P_{i}=\frac{x_{i}-x_{\min}}{x_{\max}-x_{\min}},\;\mathrm{Negative}\mathrm{indicator}:P_{i}=\frac{x_{\max}-x_{i}}{x_{\max}-x_{\min}}$$

*P_i_* is the standardized value of *i*-th index; *X_i_* is the original value of *i*-th index; *x*_max_ is the maximum value of *i*-th index; and *x*_min_ is the minimum value of *i*-th index.

Second, the “entropy weight method” is used to determine the index weight. The entropy method can not only overcome the arbitrariness caused by subjective assignment methods (Delphi method, expert scoring method, analytic hierarchy process) but also overcome the attribute repetitiveness caused by too many indicators in the complex system. It is suitable for the evaluation of objective data and diversified comprehensive indicators [34].

Finally, based on data standardization and weight calculation, we multiplied the weight of each indicator and added its standardized value to evaluate the *QOL* and the three dimensions of health, participation, and security in each provincial unit. The calculation is as follows:

(2) $$QOL=\sum_{j=1}^{n}w_{j}\times X_{ij}$$

*QOL* is the quality of life of the oldest-old in each provincial administrative unit; *X_ij_* is the standardized value of index *j* of the *i*-th provincial administrative unit; *j* = 1, 2, …; *n* is the total number of regional units; and *W_j_* is the weight of index *j*.

#### 2.4.3. Coefficient of Variation

We used the coefficient of variation to measure the regional difference of geographical things in space, which is helpful for comparing the degree of data dispersion and differentiation. The larger the coefficient of variation, the stronger the difference, and vice versa [35].

#### 2.4.4. Panel Data Model

We used a panel data model to analyze the influencing factors of QoL of the oldest-old. Compared with a cross-sectional data model and a time series model, a panel data model can enlarge sample information, reduce collinearity between variables, improve the degree of freedom and effectiveness, avoid estimation bias caused by unobservable variables (individual effects such as reproductive culture, folk customs, and legal systems), and obtain more accurate estimation results [36]. A panel data model includes a fixed-effect model and a random-effect model. Their difference is that a fixed-effect model does not need to assume that individual effects do not correlate with other explanatory variables, while a Hausman test can meet above requirement. Based on STATA14.1, the Hausman test result shows that *p* = 0.0009, which rejects the null hypothesis. Therefore, the fixed-effects model is considered more appropriate.

#### 2.4.5. Data Sources

This paper uses 31 provinces (provinces, municipalities, and autonomous regions) in mainland China as evaluation units (excluded are Taiwan Province, Hong Kong Special Administrative Region, and Macao Special Administrative Region). Considering the implementation of Active Aging policy framework in 2002 and the data availability, we choose the 2005–2015 as our study period. Data include life expectancy, health status, medical and health care, social participation, education access, and social security. Among them, the participation rate of basic medical insurance for urban employees and the number of medical and health technical personnel per 100 oldest-old individuals are from the “China Statistical Yearbook” (2006–2016). The participation rate of basic pension insurance for urban employees, the urban employees’ basic pension insurance benefits, and the socialized pension payment rate are from the “China Labor Statistics Yearbook” (2006–2016). The number of old-age activity centers per 1000 older adults, the number of old-age organizations per 1000 older adults, the number of geriatric associations per 10,000 older adults, the old-age school enrollment rate, the old-age subsidy coverage rate, and the number of old-age service beds per 1000 oldest-old come from the “China Civil Affairs Statistical Yearbook” (2006–2016). The disability, the loss-of-spouse, the average years of education, and the retirement pension’s coverage rates are from the “National 1% Sample Survey Data” (2005–2015). All the above indicators are obtained through summary and simple processing; however, the old-age subsidy coverage rate is approximated from data in 2006 and 2011 due to the lack of data in 2005 and 2010. The number of old-age activity centers, old-age organizations, and geriatric associations were approximated using data from 2006.

## 3. Results

### 3.1. Single-Dimensional Measurement

**Figure 2:**
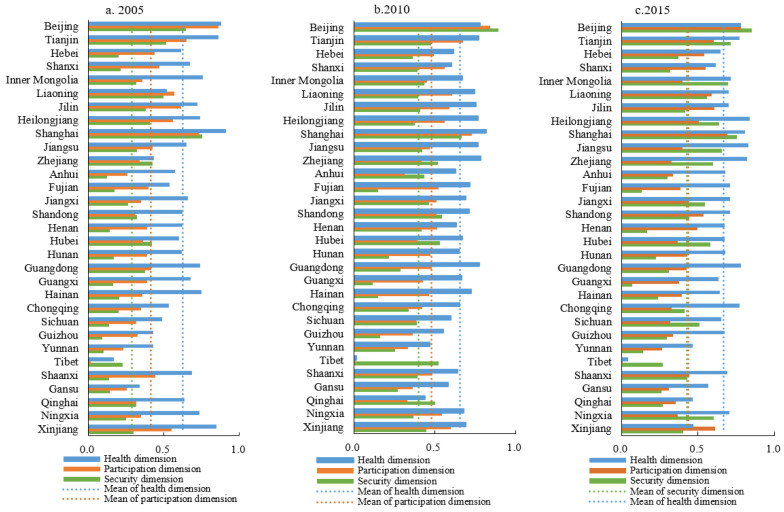
Measurement results of quality of life for the oldest-old in China, 2005–2015.

From 2005 to 2015, the health quality of the oldest-old in China improved by 6.06%. Among the provinces (municipalities directly under the Central Government rose rapidly) that rose rapidly were Zhejiang, Guizhou, and Chongqing, and some areas showed a downward trend (Xinjiang, Qinghai, and Tibet) (Figure 2). For the four major regions, the health quality in the eastern, western, and northeastern regions increased by 7.65%, 1.50%, and 14.10% respectively, and the health quality in the central region decreased by 0.33%. The overall quality of participation of the oldest-old increased by 5.64%. Among the provinces, 19 autonomous regions and municipalities including Shandong, Henan, and Hebei trended upward, while Tibet, Beijing, and Heilongjiang trended downward. While there was a 16.65% increase in participation quality in the central region, the participation quality in the eastern, western, and northeast regions decreased by 0.23%, 6.00%, and 9.94%, respectively. The QoL of the oldest-old in China has greatly improved, and the overall security quality has increased by 47.48%. The degree of improvement in security quality in different regions was led by the central region (92.52%), followed by the northeastern (56.76%), eastern (16.01%), and western (11.33%) regions. On the whole, under the guidance of the “Active Aging” theory and policy, the QoL of the oldest-old in China has improved significantly in the three dimensions of health, participation, and security, but regional imbalances still exist, and the QoL of health and participation still leave room for improvement.

① From 2005 to 2015, the areas with high health quality were mainly concentrated in the core provinces (municipalities) of the national-level urban agglomerations, which have strong financial foundations, advanced scientific research and medical technology, and abundant human resources. These agglomerations include Shanghai, Beijing, Tianjin, Zhejiang, Jiangsu, and Guangdong (Table 2). The overall coefficient of variation decreased by 2.99% during this period. Except for the unchanged coefficient of variation in the central region, the eastern, western, and northeastern regions decreased by 3.76%, 7.49%, and 6.82%, respectively. This shows that the overall difference in the health quality among the oldest-old is gradually shrinking, but the differences between regions are still significant. ② The spatial pattern of participation quality shows that the quality of participation of the oldest-old in Yunnan, Gansu, Tibet, Qinghai, and other provinces (autonomous regions) is low due to factors such as uneven distribution of regional education resources, limited investment in education funds, and insufficient vitality of the public cultural industry. During the period, the overall coefficient of variation decreased by 2.78%, the central region decreased by 8.00%, and the eastern, western, and northeastern regions increased slightly, which indicates that there were significant regional differences in the participation quality of the oldest-old. While the central regional differences have narrowed, the differences in the eastern, western, and northeastern regions have widened. ③ The high-value areas of security quality include Beijing, Shanghai, Tianjin, Jiangsu, Zhejiang, and other eastern coastal provinces (municipalities) with high urbanization rates and high employment rates. Low-value areas are mainly distributed in Guangxi and Yunnan in the southwest and Hunan, Fujian, and Henan in the central area. The large population base, the limited coverage of social insurance, and insufficient investment in pension funds are the main reasons for the relatively backward QoL in these areas. During the period, the overall coefficient of variation decreased by 21.44%, and the decreasing amplitude of the coefficient of variation of the four regions was 38.76% in the middle, 25.19% in the northeast, 7.70% in the east, and 5.51% in the west. This shows that the regional differences in security quality for the oldest-old are shrinking simultaneously as are the differences within each region.

It is worth noting that although there are regional similarities in the QoL in different dimensions, some provinces (municipalities) have obvious differences between the three dimensions, and the QoL is internally imbalanced. For example, the health quality in Jiangxi, Guangxi, Guangdong, Chongqing, and Guizhou is significantly higher than that of the other two dimensions. An internal imbalance in QoL has become an obstacle to improving the QoL. Figuring out how to balance all dimensions is key to improving QoL in the future.

### 3.2. Comprehensive Measurement

From 2005 to 2015, the QoL of the oldest-old in China improved steadily. The quality-of-life index increased from 0.5595 to 0.5833, for an increase of 4.25%. The increase in 2010–2015 (2.26%) was greater than that in 2005–2010 (1.95%), indicating that the QoL of the oldest-old is accelerating (Table 3).

From the overall situation of the four regions, the quality-of-life index in the eastern region is the highest, followed by the central region; the northeast and western regions are the lowest, showing a spatial pattern of “east-central uplift, west-northeast collapse”. During the study period, the quality-of-life index in northeast China increased significantly, from 0.5637 in 2005 to 0.6456 in 2015 for an increase of 14.53%. The eastern region also showed a slight increase (5.00%); the QoL in the central and western regions decreased by 0.84% and 3.58%, respectively. The spatial imbalance in the QoL of the oldest-old in China is prominent, and polarization still exists. For example, the QoL in Tibet, Qinghai, Gansu, Yunnan, Guangxi, Guizhou, and other provinces (autonomous regions) is still lower than the national average.

**Figure 3:**
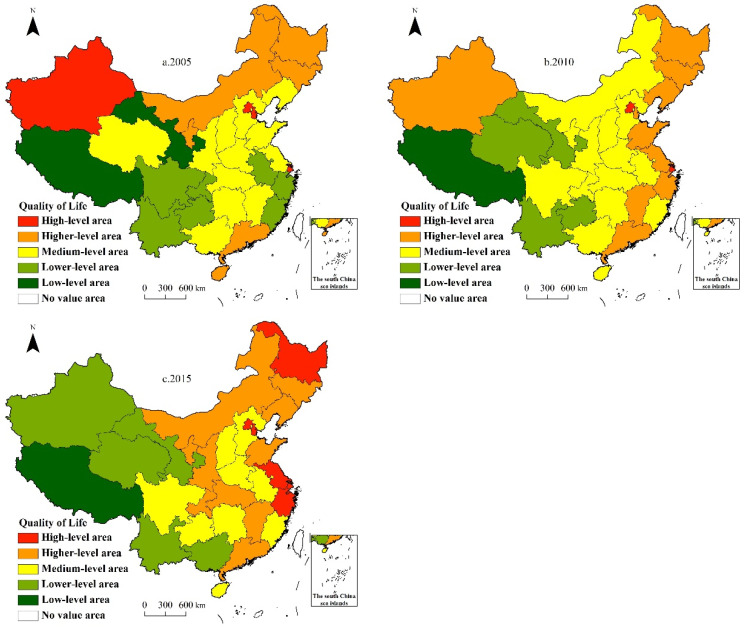
Comprehensive measurement of quality of life for the oldest-old in China, 2005–2015. Note: Standard map drawing based on the standard map service system of the Ministry of Natural Resources (the drawing number is GS (2019)1833). The base map is not modified.

We used the natural breaking point method to divide the quality-of-life index of each province into high-level areas, higher-level areas, medium-level areas, lower-level areas, and low-level areas (Figure 3). The analysis found that: ① The advantage of the middle- and higher-level areas is obvious. In 2005, 18 provinces in China were in areas with medium and high QoL, and accounted for 58.1% of all provinces. In 2010, the number rose sharply to 24 (77.4%), an increase of 33.3%. The number began to fall in 2015, dropping to 19 (61.5%), a decrease of 26.3%, but these 18 provinces still represented the vast majority of provincial units in China. This means that the improvement of the QoL of the oldest-old in China has achieved remarkable results. ② There were increases and decreases in QoL at all provincial levels. From 2005 to 2010, 32.3% of the provinces (Gansu, Anhui, Fujian, Sichuan, Chongqing, Jiangxi, Shandong, Liaoning, Jiangsu, and Zhejiang) moved to higher grades, 19.4% of provinces (Qinghai, Hainan, Mongolia, Ningxia, Xinjiang, and Tianjin) shifted to lower grades, and 48.4% of the provincial grades remained unchanged. The transfer path of different provinces is complicated. Zhejiang Province is the only province demonstrating the characteristics of leapfrogging up the levels of improvement. From 2010 to 2015, 32.3% of the provincial units (Guizhou, Mongolia, Ningxia, Shaanxi, Hubei, Chongqing, Tianjin, Heilongjiang, Jiangsu, and Zhejiang) moved to higher grades, 6.5% to lower grades (Guangxi and Xinjiang), and 61.3% of the provinces tended to be stable. Xinjiang is the only province that has shifted from higher to lower by leaps. ③ Temporal and spatial evolution characteristics show that from 2005 to 2015, the high-level areas of Beijing and Shanghai became more stable. As the two extreme centers of North and South China, Beijing and Shanghai have assumed the engine function of national economic development and promoted the improvement of the QoL of the oldest-old in the region under spillover effects. During this period, the higher-level areas gradually expanded to the eastern coastal and central inland areas, advanced along the two axes of “Heilongjiang–Liaoning–Jilin–Tianjin–Shandong–Jiangsu–Zhejiang–Jiangxi–Guangdong” and “Mongolia–Ningxia–Shaanxi–Chongqing–Hubei–Jiangxi–Guangdong in 2010 and 2015. The medium-level area occupies half of the country, but it has shown a concentration trend since 2015, and the concentration dispersed, eventually forming two major areas: “Hebei–Shanxi–Henan–Anhui” and “Sichuan–Guizhou–Hunan”. The lower-level area transits from southeast and southwest to northwest, and finally forms a low-value agglomeration belt with a southeast-northwest trend, extending from the Junggar Basin through the Tarim Basin and Qinghai-Tibet Plateau to Yunnan-Guizhou Plateau. The low-level area is relatively stable in the Tibet Autonomous Region.

### 3.3. Analysis of Influencing Factors

The estimated results showing that *F* statistics is 5.495, *p* value = 0.000 (<0.001), indicating the model’s overall fitting effect is good (Table 4). According to Table 4, the QoL of the oldest-old positively correlated with the old-age dependency ratio (ODR), the average household size (AHS), and the per capita years of education (AYE). While the QoL negatively correlated with the proportion of the oldest-old in older adults (POO), the proportion of the output value of secondary and tertiary industries in GDP (PST), and the social assistance expenditure per capita (SAE).

(1) The old-age dependency ratio (ODR) was significantly positively associated with the QoL, which was completely consistent with our previous hypothesis. The possible reason is that the higher the old-age dependency ratio in the region, the higher the aging degree, and the regions with higher aging degree usually have better socioeconomic conditions and higher quality of laborers, which can provide more social resources, pension care services, medical facilities, improve the health and participation quality of the oldest-old, and then improve their quality of life.
(2) The average household size (AHS) was significantly positively associated with the regional QoL, which was consistent with our hypothesis. Family size, as a guarantee for intergenerational economic support, life care, and spiritual comfort, is the core foundation of family care for older adults and plays an outstanding role in improving their QoL and subjective well-being [37]. The “family support theory” also contends that older adults living with their children are more likely to receive daily care and timely assistance in cases of illness or emergency [38]. However, with the implementation of the “one-child” policy, the family size in China has changed from traditional direct or joint extended families to nuclear small and empty nest families. The miniaturization of family size weakens the function of family financial support and services, and reduces the QoL and subjective well-being of older adults [39].
(3) Per capita years of education (AYE) was significantly positively associated with regional QoL. Areas with higher AYE also have higher education levels for older adults, and education can promote health through two pathways. On the one hand, a higher education level means a better job and a higher income, which is conducive to improving health investment in health care and nutrition status of the older adults. On the other hand, a higher education level means stronger cognition and health awareness, and more health knowledge can be acquired through developing good living habits, thus improving the quality of life [40].
(4) Per capita social assistance expenditure (SAE) was negatively associated with regional QoL, which is consistent with our hypothesis. The possible reason lies in the fact that the current level of social assistance in China is generally low, and the assistance standards lack uniformity and standardization. The oldest-old, especially the poor, disabled, and sick, face a lack of material life information, daily care and nursing, and targeted service aid measures [41]. In addition, older adults are a socially disadvantaged group. The provision of social assistance increases the psychological burden on the oldest-old to a certain extent, widens the gap between them and other populations, and hinders the improvement of their QoL.
(5) Contrary to our previous assumptions, the proportion of the oldest-old in the older adults (POO) and the proportion of the output value of secondary and tertiary industries in GDP (PST) were significantly negatively correlated with the QoL. ① For the POO, one possible reason is that the health, participation, and security development levels that constitute the improvement of QoL are not in harmony with the growth of the number of the oldest-old. The social medical security, endowment insurance, health services, old-age subsidies, and infrastructure for community activities for the oldest-old are insufficient to meet the basic needs of the growing number of the oldest-old. Take the regional old-age subsidy as an example. In 2015, the proportion of the oldest-old in the eastern region was higher than that in the western region, but the standardized average of the old-age subsidy coverage rate (1.288) lagged behind that of the western region (2.860). This was mainly because the poverty problem in the western region was more severe than that in other regions; thus, the old-age subsidy, which was a welfare program for the oldest-old, played a more important role in poverty relief in the western region, and the age restrictions were more relaxed [42]. For example, Shaanxi and Qinghai provinces, where the coverage rate of the old-age subsidy exceeds 100%, target individuals aged 70 and above. In terms of people’s livelihoods, the old-age subsidy system has become the primary choice for western provinces to enhance their competitiveness, and the state has corresponding policy preferences for these areas [42]. ② For the PST, the secondary and tertiary industries are usually used to measure the overall economic strength and modernization degree of a region, reflecting society’s ability to absorb employment. However, in the context of the miniaturization of the family, the huge supply gap in the social pension service industry, and the extent of the domestic service industry, the secondary and tertiary industries result in a lack of care resources or an insufficient supply of care services for the offspring of families. The old-age care service industry is an emerging industry formed by the needs of older adults in the consumption market. It is still in its infancy, stagnating in terms of pension economic sources, service providers, service content, and level of care, especially in the developed eastern regions where the oldest-old population is growing rapidly. How to balance and coordinate the contradiction between the old-age service industry and the growing demand of the oldest-old has become an important concern for protecting people’s livelihoods in the new era.

## 4. Conclusions and Discussion

Our results show that the overall QoL of the oldest-old has steadily improved from 2005 to 2015, and the QoL improved significantly in all dimensions. While uneven and insufficient regional development still exists, the quality of health and participation remain to be improved. 

The influencing factor results show that the per capita years of education, the old-age dependency ratio, and the average household size have an impact on the QoL of the oldest-old through the effects of medical health, consumption, and family care, which is in line with the research assumptions. ① The role of education in promoting health can be attributed to the “relaxation of budget constraints theory” and “resource allocation efficiency theory”. “Relaxation of budget constraints theory” holds that a higher education level means better jobs and higher incomes, which is conducive to improving the health investment of individuals in medical care and nutritional conditions [43]. “Resource allocation efficiency theory” believes that education can indirectly change people’s cognition and behavior of health and promote the “allocation efficiency” and “production efficiency” of health. ② Regions with a higher old-age dependency ratio provide more social resources and old-age services for the oldest-old through good economic conditions and high-quality labor force, so as to improve the health and participation quality. ③ The influence of the declining average household size on QoL requires us to give attention to the family care and nursing of the oldest-old to ensure QoL for the oldest-old in their later years.

Based on the above research conclusions, we propose several countermeasures and suggestions for improving the QoL among the oldest-old in China. ① First, in view of the uneven and insufficient regional development of QoL at this stage, the coordination of regional economic and social development should be a priority for the government [2]. ② Second, accelerate the formulation of family pension support policy system and promote the family pension function, to make up for the lack of economic support and service provision caused by family miniaturization. For example, in terms of family service provision, expanding the subject (currently mainly the staff of state organs, enterprise, and public institutions) of family leave system, and increasing the family leave time for married children can improve the opportunity for the oldest-old to enjoy family care services. In terms of economic support, tax incentives and subsidies should be adopted. For example, young people who live together with the oldest-old will be given tax breaks or rent or purchase subsidies to meet the needs of the oldest-old for family care. ③ Third, improve the social old-age services supply and establish a medical security system covering urban and rural oldest-old. Furthermore, strengthen the community intervention service function and build a diversified community home-care model. Relying on big data to build a smart elder-care service platform, provide the oldest-old with digital services such as online and offline professional medical treatment, rehabilitation, health care, nursing, and entertainment [44].

Under the increasingly severe situation of “aging before getting rich” and “aging before getting ready”, evaluating the QoL of the oldest-old from the perspective of the Active Aging policy framework provides a new perspective for effectively responding to a post-aging society. This perspective breaks through the previous paradigm of life quality research, trying to incorporate the concepts of group development, harmony, and sharing at the individual level, creating a positive atmosphere to encourage the oldest-old to participate in society and make social contributions to ensure their independence, self-realization, and dignity in their later years. At the same time, life quality studies from a spatial perspective not only meet the multidimensional demands of QoL but also reflect the comprehensive, regional, and interdisciplinary characteristics of geography. These studies are designed to improve the group QoL evaluation system and enrich the perspective of population geography research. Promoting the cross-interaction of population geography provides an interdisciplinary platform. However, several limitations should be noted when conducting our research. First, restricted by the availability of data at the national level, the selection of some indicators lacks the pertinence of the research group. Discovering how to use the local characteristics of geography to explore feasible elements that meet the quality of specific group needs to be deepened in the future. Second, based on the “Active Aging Framework”, this paper constructed a localized quality-of-life assessment system. Whether this framework can be promoted globally is worth exploring. In the future, we could use comparable indexes such as Active Aging Index (AAI) to better understand the situation about the oldest-old on a global level and enrich our findings.

## Figures and Tables

**Table 1:** Evaluation index of quality of life for the oldest-old in China.

Target Layer (A)	Criterion Layer (B)	Index Layer (C)	Unit	Index Nature	Symbol
Quality of life (A)	Health dimension (B1)	C1: The average life expectancy	Year	Positive	ALE
		C2: The disability rate of older adults	%	Negative	DR
		C3: The spouse rate of older adults	%	Positive	SR
		C4: The participation rate of the basic medical insurance for urban employees	%	Positive	UBMI
		C5: The number of medical and health technical personnel per 100 oldest-old	People	Positive	PMHT
	Participation dimension (B2)	C6: The average years of education of older adults	Year	Positive	AYE
		C7: The number of old-age activity centers per 1000 older adults	%	Positive	SAC
		C8: The number of old-age organizations per 1000 older adults	%	Positive	OAO
		C9: The number of geriatric associations per 10,000 older adults	Number	Positive	OAA
		C10: The old-age school enrollment rate of older adults	%	Positive	OAS
	Security dimension (B3)	C11: The old-age subsidy coverage rate	%	Positive	OSC
		C12: The coverage rate of older adults’ retirement pension ^1^	%	Positive	PCR
		C13: The urban pension insurance participation rate	%	Positive	UBPI
		C14: The number of old-age service beds per 1000 older adults	Number	Positive	BOC
		C15: The urban employees’ basic pension insurance benefits	10,000 RMB	Positive	BPB
		C16: The socialized pension payment rate ^2^	%	Positive	SPP

^1^ The coverage rate of older adults’ retirement pension: refers to the proportion of the older adults aged 60 and above whose main source of living is retirement pension in a region to the total number of older adults aged 60 and above in that region. ^2^ The socialized pension payment rate: refers to the proportion of the number of older adults aged 60 and above who receive social pension in a region.

**Table 2:** The coefficient of variation of health, participation, and security quality of life for the oldest-old.

	Health Quality (%)			Participation Quality (%)			Security Quality (%)		
	2005	2010	2015	2005	2010	2015	2005	2010	2015
National	1.26	1.24	1.23	1.56	1.44	1.52	1.86	1.58	1.53
East	1.19	1.16	1.15	1.34	1.28	1.35	1.52	1.45	1.41
Central	1.18	1.21	1.18	1.61	1.43	1.49	1.83	1.21	1.32
West	1.33	1.30	1.32	1.72	1.58	1.77	1.73	1.55	1.64
Northeast	1.21	1.14	1.14	1.34	1.33	1.42	1.85	1.65	1.48

**Table 3:** Descriptive statistics of quality-of-life index for the oldest-old in China.

Year	National Average	Maximum	Minimum	Eastern Average	Central Average	Western Average	Northeast Average
2005	0.5595	0.8662 (Shanghai)	0.1521 (Tibet)	0.6395	0.6705	0.5414	0.5637
2010	0.5704	0.8147 (Beijing)	0.1163 (Tibet)	0.6626	0.6552	0.5226	0.6450
2015	0.5833	0.7943 (Beijing)	0.0804 (Tibet)	0.6715	0.6649	0.5220	0.6456

Note: In parentheses are the abbreviations of corresponding provinces, autonomous regions, and municipalities directly under the Central Government.

**Table 4:** Results of model estimation.

Variables	Coefficient	Standard Error	*T* Value	*p* Value	95% Confidence Interval
Constant	−0.283	0.372	−0.76	0.450	[−1.028, 0.462]
*POO*	−0.013 ***	0.004	−3.61	0.001	[−0.020, −0.006]
*ODR*	0.022 ***	0.007	3.15	0.003	[0.008, 0.035]
*AHS*	0.105 *	0.059	1.79	0.080	[−0.013, 0.224]
*lnGDP*	−0.060	0.052	−1.16	0.253	[−0.163, 0.044]
*PST*	−0.005 **	0.003	−2.12	0.038	[−0.011, 0.000]
*UL*	0.005	0.003	1.36	0.179	[−0.002, 0.011]
*UR*	−0.021	0.019	−1.06	0.295	[−0.060, 0.018]
*AYE*	0.163 ***	0.039	4.17	0.000	[0.085, 0.242]
*SAE*	−0.001 **	0.000	−2.06	0.044	[−0.001, 0.000]
Mean dependent var		0.571		SD dependent var	0.137
R-squared		0.483		Number of obs	93.000
*F*-test		5.495		Prob > *F*	0.000
Akaike crit. (AIC)		−332.960		Bayesian crit. (BIC)	−307.634

*** *p* < 0.01, ** *p* < 0.05, * *p* < 0.1.

## Data Availability

We obtained the data from China Statistical Yearbook 2006–2016, China Labor Statistics Yearbook 2006–2016, China Civil Affairs Statistical Yearbook 2006–2016 and National 1% Sample Survey Data 2005–2015.
